# Expression response of chalcone synthase gene to inducing conditions and its effect on flavonoids accumulation in two medicinal species of *Anoectochilus*

**DOI:** 10.1038/s41598-019-56821-0

**Published:** 2019-12-27

**Authors:** Lin Yang, Jun Cheng Zhang, Jing Tao Qu, Gang He, Hao Qiang Yu, Wan Chen Li, Feng Ling Fu

**Affiliations:** 1 Maize Research Institute, Sichuan Agricultural University, Chengdu, Sichuan 611130 PR China; 2 Medical Plant Exploitation and Utilization Engineering Research Center, Fujian Province University, Sanming University, Sanming, 365004 People’s Republic of China; 3 Key Laboratory of Medicinal and Edible Plants Resources Development of Sichuan Education Department, Sichuan Industrial Institute of Antibiotics, Chengdu University, Chengdu, 610052 PR China

**Keywords:** Gene expression, Plant physiology

## Abstract

*Anoectochilus roxburghii* and *Anoectochilus formasanus* are the major species of genus *Anoectochilus* used in traditional Chinese medicine for their abundant content of flavonoids and some other medicinal constituents. In recent years, their wild resources are gradually exhausted due to over-collection and ecological deterioration. Artificial cultivation and tissue culture are employed to increase production. In this study, the open reading frame, promoter and genomic sequences of the chalcone synthase (CHS) gene were cloned from these two species according to their transcriptome information, and used for expression analysis in response to the induction of phenylalanine, ultraviolet light and NaCl, and its effect investigation on accumulation of flavonoids. The results showed that the expression of the *CHS* genes was upregulated in response to these inductions and resulted in increasing accumulation of total flavonoids. However, the increased flavonoids induced by phenylalanine and ultraviolet light were mainly allocated into the anthocyanidin branch of flavonoids biosynthesis. Not only did it improved the medicinal value, but might have inhibitory effect on plant growth because of the increased malondialdehyde accumulation. Under the induction of appropriate concentration of NaCl, the medicinal constituents of flavonoids were increased without inhibition to plant growth.

## Introduction

*Anoectochilus roxburghii* and *Anoectochilus formasanus*, native to Fujian and Taiwan provinces of China respectively, are the major species of genus *Anoectochilus* of family Orchidaceae, used as herbal drug and health food in traditional Chinese medicine^1^.They are used to treat tumor, cardiovascular disease, fatty liver, hepatitis and other diseases because of their abundant medicinal constituents such as flavonoids and polysaccharides^2–7^. Due to overharvesting and ecological deterioration, their wild populations have been gradually exhausted, and a lot of effort has been made for artificial cultivation and tissue culture for rapid propagation^8–10^. However, the accumulation of the medicinal constituents in the cultivated plants may be different from the wild plants. It becomes important to investigate the effect of cultivation or tissue culture conditions to the accumulation of the medicinal constituents, as well as its molecular mechanism^10–14^.

Flavonoids are a large category of aromatic secondary metabolites ubiquitous in plant and fungus. These compounds include six major subgroups that are found in higher plants: the chalcones, flavones, flavonols, flavandiols, anthocyanins, and condensed tannins (or proanthocyanidins); a seventh group, the aurones, is widespread, but not ubiquitous^15,16^. Most of these constituents have antioxidant properties^17^. The flavonol subgroup, including rutin (rutinoside), quercetin and kaempferol, is the major medicinal constituents of *A*. *roxburghii*and *A*. *formasanus*^1,18,19^. In plants, flavonoids are biosynthesized through phenylpropanoid pathway from initial substrate phenylalanine (Phe). Chalcone synthase (CHS) catalyzes the conversion of 4-coumaroyl-CoA and 3 units of malonyl-CoA to chalcone, 4 nuits of coenzyme A (CoA-SH) and carbon dioxide (CO_2_). This is the committed step branching to biosynthesis of flavonol, isoflavone and anthocyanidin (Fig. S1)^16,20^. The *CHS* gene has been cloned and characterized from *Phalaenopsis hybrid*^21^, a relative species of another genus of family *Anoectochilus*, as well as many other plants^22–27^. Its expression is responsive to induction of Phe^28,29^, ultraviolet light (UV)^11,30–33^, NaCl^11,34,35^ and some other biotic and abiotic conditions.

However, genomic information has not been available for any species of genus *Anoectochilus*, and even there are few reports about their chromosome ploidy^36^. It is difficult for homologous cloning of genes from any species of this genus. In the present study, transcripts were assembled from RNA sequencing (RNA-seq) data, and used to clone the open reading frame (ORF) and the genomic sequences of the *CHS* gene from *A. roxburghii* and *A. formosanus*. After function evaluation by bioinformatics analysis, subcellular localization, enzyme activity assay and heterologous expression, their expression response to induction of Phe, UV, and NaCl was measured by real-time quantitative PCR (RT-qPCR).Their promoter sequences were amplified by thermal asymmetric interlaced PCR (TAIL-PCR) and used for prediction of *cis*-acting elements. The contents of total flavonoids, free radical scavenging activity (FRSA), content of anthocyanidin and flavonols (rutin, quercetin, and kaempferol), as well as malondialdehyde (MAD), were measured by spectrophotometry and high performance liquid chromatography (HPLC), respectively. The possibility was explored to upregulate the expression of the *CHS* gene and to increase the accumulation of total flavonoids or flavonols in *A. roxburghii* and *A. formosanus* by induction in artificial cultivation or tissue culture.

## Results

### The CHS genes and their putative proteins

8.76 and 10.42 Gb raw bases, and 29213781 and 34742855 raw reads were obtained by RNA-seq of the *A. roxburghii* and *A. formosanus* samples. Through quality inspection and filtration, 8.28 and 9.85 Gb clean bases, and 27597990 and 32836885 clean reads were retained, respectively, with Q20 (sequence error rate below 1%) greater than 96% and Q30 (sequence error rate below 0.1%) greater than 90% (Fig. S2). From these clean reads, 130024 and 116423 transcripts were assembled, 30265 and 24425 of them were annotated by SWSSPROT, and 35176 and 28691 of them were annotated by KGO, respectivly. One of them was annotated as the *CHS* gene for *A. roxburghii* and *A. formosanus*, respectively. These two transcripts shared 94.80% similarity (Fig. S3).

**Figure 1:**
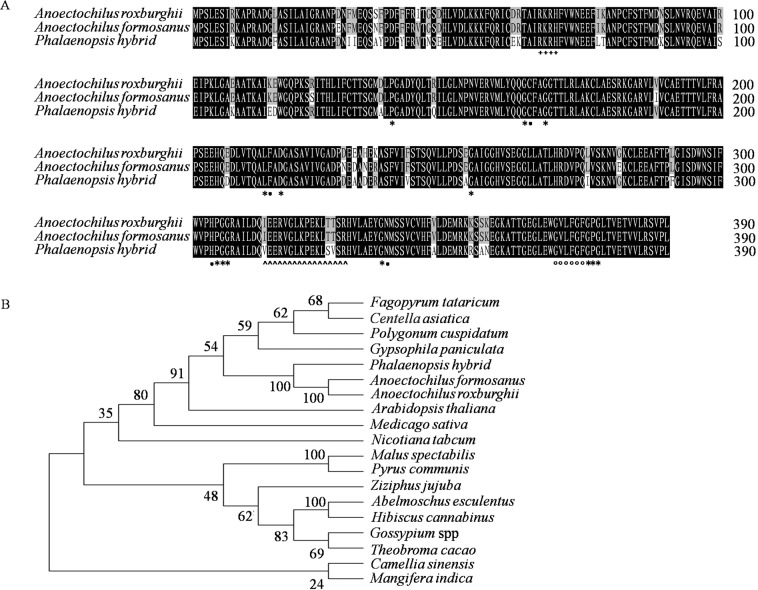
Alignment and phylogenetic tree of putative proteins of gene *CHS* of *A. roxburghii* and *A. formosanus* with relative species. (**A**) Alignment among the putative proteins of gene *CHS* of *A. roxburghii*, *A. formosanus* and *P. hybrida*. The black, gray and white backgrounds represent similarity of 100%, 66.7% and 0%, respectively. The asterisks (*), the black dots (•), the hollow dots (○), the hollow triangles (△) and the plus (+) represent the activation sites, the catalytic residues, the conserved residues, the malonyl-coenzyme A binding motif and the nuclear localization signal, respectively. (**B**) Phylogenetic tree among the putative proteins of gene *CHS* of 19 relative species.

With the primers homologous to the assembled transcripts, specific fragments of more than 1000 bp were amplified from the cDNA and the genomic DNA samples of *A. roxburghii* and *A. formosanus*, respectively (Fig. S4). The sequence analysis showed that their genomic fragments were both 1260 bp long and shared similarity of 94.92%. Their ORFs (1173 bp) were interrupted by an intron of 87 bp from the 180th to the 267th bp (Fig. S5). Their putative proteins (390 aa) were highly homologous (90.75% and 90.00%) with that of the *CHS* gene (AAV70116.1) in *P. hybrida* of family *Anoectochilus* (Fig. 1A)^21^. The predicted molecular weight (42.9 kDa), isoelectric points (pI 6.04 and 6.24), grand averages of hydropathicity (−0.116 and −0.123), secondary structure (31.54% and 32.82% α-helices, 21.54% and 20.51% extended strands, 46.92% and 46.67% random coils, respectively), and three three-dimensional structure models were all similar to that (42.5 kDa, pI 6.04 and −0.057, 29.74% α-helices, 21.03% extended strands, and 49.23% random coils) of the putative CHS protein in *P. hybrida* (Fig. S6)^21^. In the phylogenetic tree, the putative proteins of these two ORFs were also clustered into the same group with the putative CHS protein in *Phalaenopsis hybrida* (Fig. 1B). All the thirteen activation sites (G^138^, G^163^, G^167^, L^214^, D^217^, G^253^, P^305^-G^306^-G^307^, G^336^, and G^385^-P^386^-G^387^), the four catalytic residues (Cys^164^, Phe^215^, His^304^ and Asp^337^) and the conserved domain of six residues (G^379^-V^380^-L^381^-F^382^-G^383^-F^384^) were the same, except the three amino acids different in the malonyl-CoA binding motif (V^314^-E^315^-E^316^-R^317^-V^318^-G^319^-L^320^-K^321^-P^322^-E^323^-K^324^-L^325^-T^326^-T^327^-S^328^-R^330^)^21^.

### Function evaluation of the CHS genes

**Figure 2:**
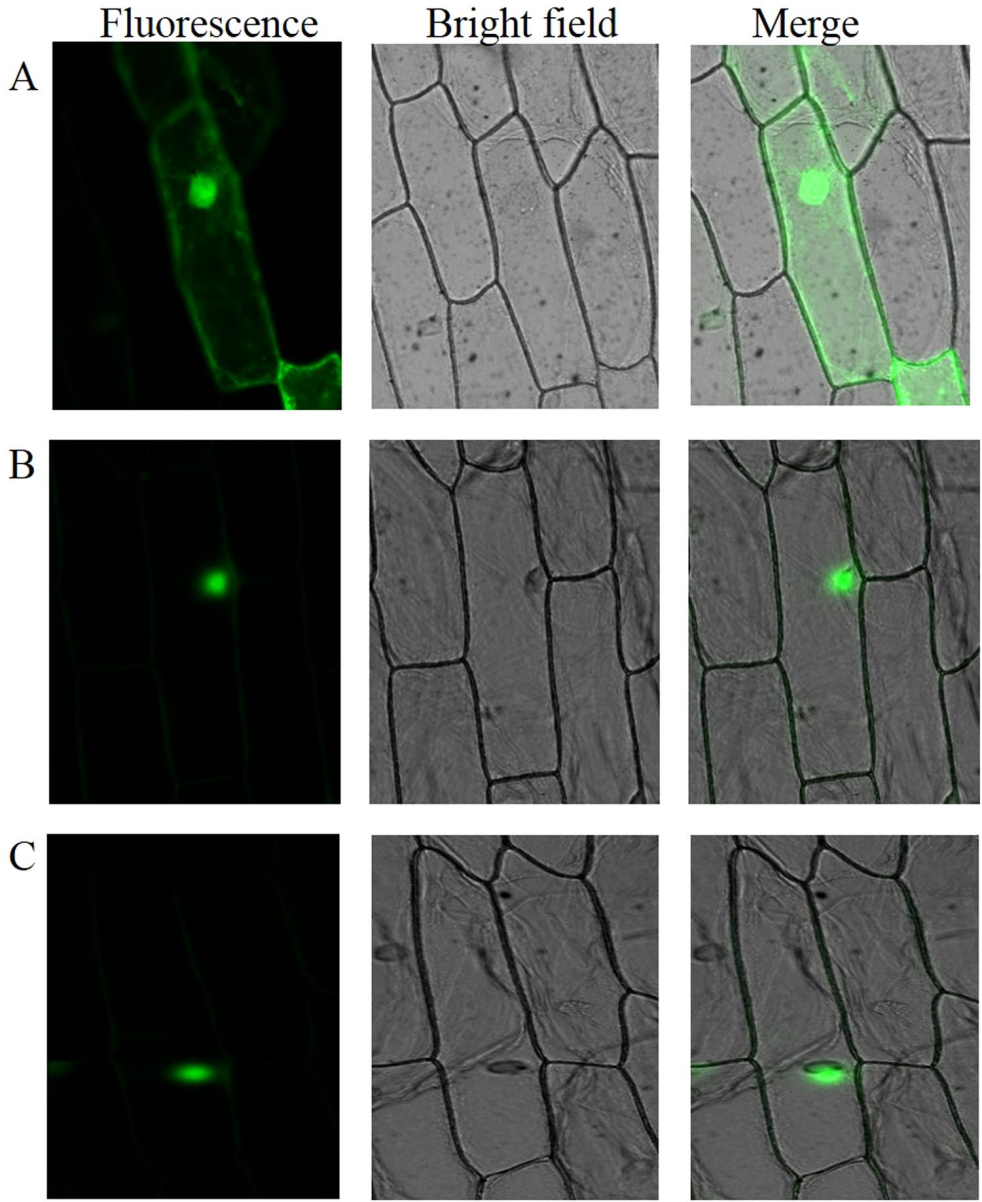
Subcellular localization of CHS protein. (**A**) Negative control transformed by empty vector pC2300-*35S*-*eGFP*. (**B**) Transient expression of vector pC2300-*35S-CHS*-*eGFP* harboring the *A. formosanus CHS* gene. (**C**) Transient expression of vector pC2300-*35S-CHS-eGFP* harboring the *A. roxburghii CHS* gene.

The green fluorescence signal was observed in the nucleus of the inner epidermis cell of onion transiently expressing vectors pCAMBIA2300-*35S*-*CHS*-*eGFP*, whereas it distributed in the whole cell infiltrated by the negative control vector pCAMBIA2300-*35S*-*eGFP* (Fig. 2). This result indicated the subcellular localization of the CHS protein in nucleus in *A. roxburghii* and *A. formosanu*.

**Figure 3:**
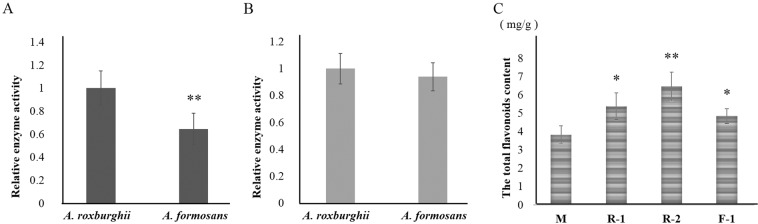
Relative enzyme activity of *E*. *coli* striansand total flavonoids content of rice lines heterologously-expressed CHS protein of *A. formosanus* and *A. roxburghii*. The asterisk (*) and the double asterisk (**) stand for significance (P ≤ 0.05) and great significance (P ≤ 0.01), respectively. (**A**) *in vitro* enzyme activity. (**B**) *in vivo* enzyme activity. (**C**) The total flavonoids contentin transgenic rice lines F-1 (transformed by the *A. formosanus CHS* gene), R-1 and R-2 (transformed by the *A. roxburghii CHS* gene).

By sodium dodecyl sulfate-polyacrylamide gel electrophoresis (SDS-PAGE), an obvious additional band about 44.3 kDa was separated from the *Escherichia coli* lines transformed by the *CHS* genes of these two medicinal species, and induced by isopropyl *β*-D-thiogalactopyranoside (IPTG) (Fig. S7). The *in vivo* CHS enzyme activities of thetransformed *E. coli*lines of the *A. roxburghii CHS* gene was significantly higher than that transformed by the *A. Formosanus CHS* gene, while the *in vitro* activities showed no significant difference (Figs. 3A,B and S8).

From the rice calli transformed by the *CHS* genes, three T_1_ lines (two of *A. roxburghii*and one of *A. formosanus*) were identified as positive by PCR amplification of the *CHS* gene fragment (Fig. S9). Their contents of total flavonoids were 1.40, 1.68 and 1.26 times higher than that of the untransformed acceptor line, respectively (Fig. 3C).

All these results confirmed that the two amplified ORF sequences were the *CHS* genes of *A. roxburghii* and *A. formosanus*, respectively, and registered at GenBank with accession numbers MK370742 and MK370743.

### Differential expression in response to inducing conditions

**Figure 4:**
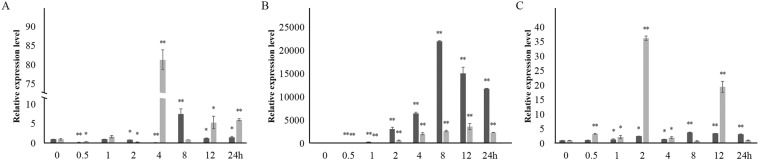
Relative expression levels of gene *CHS* of *A. roxburghii* and *A. formasanus* under Phe, UV and NaCl induction. The lighter and the darker columns represent *A*. *roxburghi* and *A*. *formosanus*, respectively. The asterisk (*) and the double asterisk (**) stand for significance (P ≤ 0.05) and great significance (P ≤ 0.01), respectively. (**A**) Phe induction. (**B**) UV induction. (**C**) NaCl induction.

Under the induction of Phe and UV, the expression of the *CHS* genes was upregulated significantly in *A. roxburghii* and *A. formosanus*, and reached its peak values (81.53 and 7.49 times of the 0 h control, respectively) at 4 and 8 h of the Phe induction, and (21994.7 and 3654.7 times of the 0 h control, respectively) at 8 and 12 h of the UV induction (Fig. 4A,B). In response to the NaCl induction, the expression of the *CHS* genes was continually upregulated and reached about three times of the 0 h control in *A. roxburghii*, while the upregulated expression reached 36.2 and 19.5 times of the 0 h control at 2 and 12 h of the NaCl induction in *A. formosanus* (Fig. 4C). This result indicated the strong expression response of these two *CHS* genes.

From the products of the second and the third rounds of thermal asymmetric interlaced PCR (TAIL-PCR) amplification from the genomic DNA samples, specific fragments were separated and sequenced for prediction of promoters and *cis*-acting elements of the *CHS* genes by Plant CARE software (Fig. S10). The promoter sequence of the *CHS* gene of *A. roxburghii* was 342 bp long containing enhance elements TATA-box (4), 5′-UTR Py-rich stretch (1) and CAAT-motif (6), light responsive elements G-box (2), *sp1*(18) and I-box (1), and anaerobic inducing element ARE (1). The promoter sequence of the *CHS* gene of *A. formasanus* was 314 bp long containing enhance elementsTATA-box (1), AT-rich sequence (1) and CAAT-box (5), light responsive elements G-box (2), CATT-motif (1), CGT-motif (5), GA-motif (1) and *sp1* (2), MeJA-responsive elementsCGTCA-motif (1) and TGACG-motif (1), and anaerobic inducing element ARE (1) (Fig. S11). This result might partially explain the expression response of the *CHS* genes to the UV and NaCl induction.

### Flavonoids accumulation under inducing conditions

**Figure 5:**
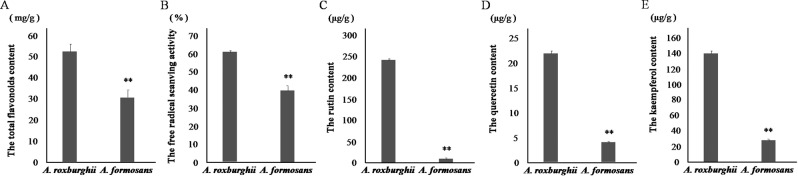
Content of total flavonoids and flavonols, and FRSA in *A. roxburghii* and *A. formosanus* under non inducing condition. The asterisk (*) and double asterisk (**) stand for significance with the control at 0.05 and 0.01 levels, respectively. (**A**) Content of total flavonoids. (**B**) FRSA. (**C**) Content of flavonols (rutin, quercetin and kaempferol).

Under non-inducing conditions (the 0 h control), the accumulation of total flavonoids, FRSA, and the accumulation of three flavonols (rutin, quercetin, and kaempferol) of *A. roxburghii* was 1.73, 1.52, 25.76, 5.48 and 6.02 times of that of *A.formoasnus*, respectively (Fig. 5), indicating its higher antioxidant activity and medicinal value.

**Figure 6:**
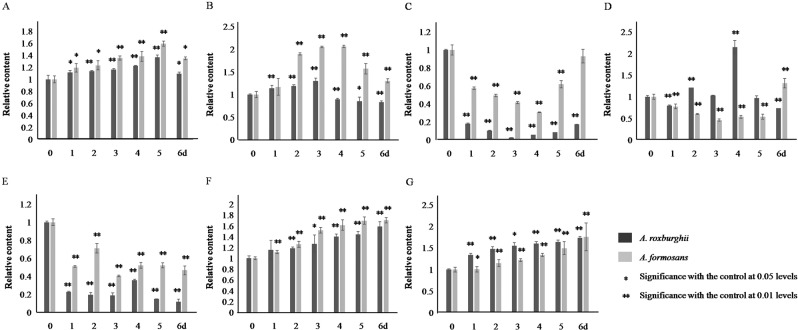
Content of total flavonoids, flavonols, anthocyanidin, and MDA, and FRSA in *A. roxburghii* and *A. formosanus*under Phe induction. The asterisk (*) and double asterisk (**) stand for significance with the control at 0.05 and 0.01 levels, respectively. (**A**) flavonoids content. (**B**) Free radical scavenging rate. (**C**) rutin content. (**D**) quercetin content. (**E**) kaempferol content. (**F**) Anthocyanidin content. (**G**) MDA content.

In response to Phe induction, the accumulation of total flavonoids and FRSA in *A. roxburghii* and *A.formoasnus* was both increased and reached their peak values (1.38, 1.51, 1.30, and 2.06 times of the 0 d control) on the 5th, 3rd, and 4th day, respectively (Fig. 6A,B). The difference of total flavonoids content was not significant between these two species, but the increased range of FRSA in *A. roxburghii* was significantly lower than that in *A. formoasnus*. The accumulation of rutin, quercetin and kaempferol was decreased during the first three days of the induction but rebounded after that with different ranges between the two species (Fig. 6C–E).The accumulation of anthocyanidin and MDA was increased continually with different ranges between the two species (Fig. 6F,G).

**Figure 7:**
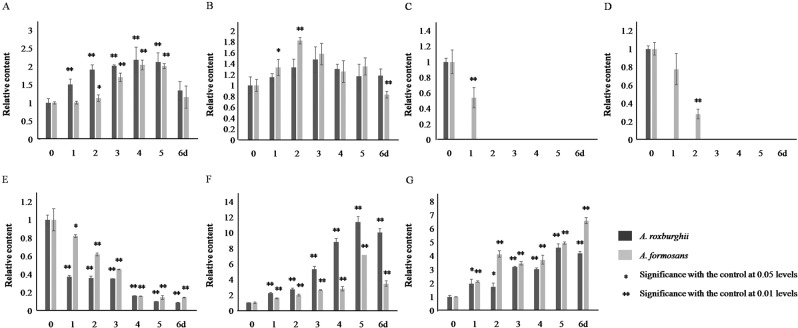
Content of total flavonoids, flavonol, anthocyanidin, and MDA, and FRSA in *A. roxburghii* and *A. formosanus* under UV induction. The asterisk (*) and double asterisk (**) stand for significance with the control at 0.05 and 0.01 levels, respectively. (**A**) flavonoids content. (**B**) Free radical scavenging rate. (**C**) rutin content. (**D**) quercetin content. (**E**) kaempferol content. (**F**) Anthocyanidin content. (**G**) MDA content.

Under UV induction, the accumulation of total flavonoids and FRSA in the two species was increased and reached their peak values (2.09, 2.01, 1.47, and 1.82 times of the 0 h control) on the 5th, 4th, 3rd, and 2nd day, respectively (Fig. 7A,B). The accumulation of rutin, quercetin and kaempferol was decreased and rapidly descend to their valley values on the 1st to 5th day (Fig. 7C–E). The accumulation of anthocyanidin and MAD was increased continually (Fig. 7F,G).

**Figure 8:**
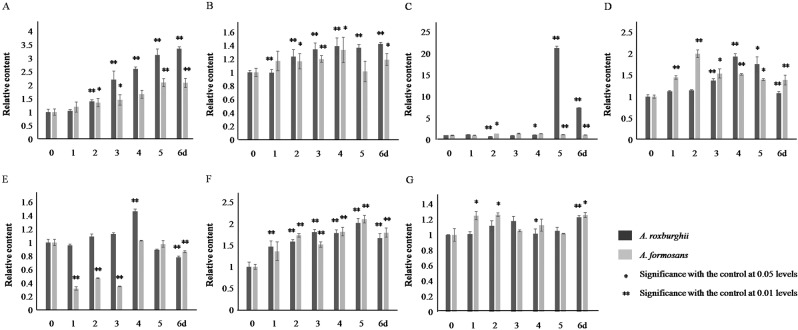
Content of total flavonoids, flavonols, anthocyanidin, and MDA, and FRSA in *A. roxburghii* and *A. formosanus* under NaCl induction. The asterisk (*) and double asterisk (**) stand for significance with the control at 0.05 and 0.01 levels, respectively. (**A**) flavonoids content. (**B**) Free radical scavenging rate. (**C**) rutin content. (**D**) quercetin content. (**E**) kaempferol content. (**F**) anthocyanidin content. (**G**) MDA content.

In response to NaCl induction, the accumulation of total flavonoids and FRSA was increased continually with different ranges between *A. roxburghii* and *A. formasanus* (Fig. 8A,B). The rutin accumulation in *A. roxburghii*was increased sharply on the 5th and 6th day and reached as high as 21.28 times of the 0 h control, whereas that in *A. formasanus* did not changed obviously (Fig. 8C). The accumulation of quercetin, kaempferol and anthocyanidin was increased with different ranges between the two species (Fig. 8D–F). The MAD content was only increased slightly (1.23 and 1.26 times of the 0 h control, Fig. 8G).

## Discussion

Numerous studies showed that the biosynthesis of flavonoids, as well as flavonols, took place exclusively in endoplasmic reticulum, cytosol, and other cytoplasmic organelles^19,36–38^. In this study, the subcellular localization of the CHS proteins of *A. roxburghii* and *A. formasanus*was targeted to the nucleus (Fig. 2). This result was also evidenced by the bioinformatics prediction of a nuclear localization signal (Arg^66^-Lys^67^-Arg^68^-His^69^) at the N-terminal of their primary structure (Fig. 1A). Some recent reports from a number of different plant species not only documented the presence of flavonoids in nuclei^39,40^, but also localized at least two of their biosynthetic enzymes to nuclei in several cell types in *Arabidopsis*^41^. It was speculated that the *Anoectochilus* CHS proteins might catalyze flavonoids synthesis of the phenylpropane metabolism pathway in nucleus, and their products might be involved not only in the basal metabolism, stress response, reproductive development and many other growth and development processes^30–35^, but also in the transcriptional regulation of related genes.

Under Phe, UV and NaCl induction, the expression of the *CHS* genes of *A. roxburghii* and *A. formasanus* were upregulated with different ranges (Fig. 4). The *cis*-acting elements related to light and anaerobic response were predicted in the promoter sequences of the two *CHS* genes (Fig. S11)^42–44^. These results not only explain the increased accumulation of total flavonoids in the two species under these inducing conditions, but also confirm the rate-limiting role of the CHS enzyme for flavonoids biosynthesis of phenylpropanoid pathway (Fig. S1)^16,20–27^.

Under Phe and UV induction, the decreased flavonols (Figs. 6C–E and 7C–E) and the increased accumulation of anthocyanidin (Figs. 6F and 7F) demonstrated that the increased accumulation of total flavonoids (Figs. 6A and 7A) was mainly allocated into the anthocyanidin branch of the phenylpropanoid pathway (Fig. S1). This was not conducive to improving medicinal value of these two species. Even more, the increased MDA accumulation revealed the inhibitory effect of Phe and UV on plant growth (Figs. 6G and 7G). Similar effect was also found in cell suspension culture of strawberry and calli of *Hydrocotyle bonariensis*^28,29^. In response to NaCl induction, the increased flavonols (Fig. 8C–E), especially the sharp peak content of rutin on the 5th and 6th day of induction in *A. roxburghii*, indicated that the increased accumulation of total flavonoids (Fig. 8A) was mostly allocated into the flavonol branch (Fig. S1), while the MAD content was only increased slightly (Fig. 8G). Along with the higher accumulation of total flavonoids, FRSA, and the accumulation of flavonolsin *A. roxburghii*under non-inducing conditions (Fig. 5), it was suggested that NaCl induction of appreciate concentration (100 mmol/L) in artificial cultivation or tissue culture of *A. roxburghii* could efficiently increase the accumulation of total flavonoids and flavonols, and improved the medicinal value.

## Conclusion

The Phe, UV and NaCl induction upregulates the expression of the chalcone synthase genes and affects the accumulation of total flavonoids, FRSA, flavonols, anthocyanidin and MAD in *A. roxburghii* and *A. formasanus*. NaCl induction of appreciate concentration (100 mmol/L) is suggested to apply in artificial cultivation or tissue culture of *A. roxburghii* to increase accumulation of total flavonoids and flavonols and improve the medicinal value.

## Materials and Methods

### Sample preparation

The seedlings of *A*. *roxburghii* and *A*. *formosanus* were cultured on MS medium for 18 weeks. The uniform seedlings were divided into three groups. Two groups were transplanted into a plastic mesh grid for aquaculture. On the fifth day, Phe and NaCl were added into the nutrient solution with final concentration of 4 mg/L and 100 mmol/L, respectively. The other group was transplanted into plastic pots (five seedlings per pot) with nutritional soil and vermiculite (3:1), and submitted to UV induction of 253.7 nm after recovering. At 0 (control), 0.5 h, 1 h, 2 h, 4 h, 8 h, 12 h, 24 h (1 d), 2 d, 3 d, 4 d, 5 d and 6 d of the Phe, UV and NaCl induction, two leaves were sampled from each of the five induced plants.

One leaf of each sample of 0 to 24 h of the induction was pulverized to fine powder in liquid nitrogen, and used for RNA extraction with RNeasy Plant Mini Kit (Qiagen, China). After release of probable DNA contamination by RNase-free DNase I (Qiagen, China), detection for concentration, purity and integrity on spectrophotometer (NanoDrop One, Thermo Fisher Scientific, USA) and Agilent 2100 Bioanalyzer (Agilent Technologies, USA) respectively, part of each RNA samples was reverse transcribed to cDNA by using PrimeScript RT Reagent Kit (TaKaRa Japan). Part of pulverized leaf samples of the control (0 h) were mixed and used for extraction of genomic DNA with the method of cetyl trimethylammonium bromide (CTAB).

### Transcriptome sequencing and CHS gene cloning

Part of the RNA samples of the control (0 h) were mixed and sequenced by Illumina HiseqXten platform at MajorbioBioTech Co., Ltd. The clean reads were assembled by Trinity v2.4.0 (https://github.com/trinityrnaseq/trinityrnaseq/wiki), and used to search for transcript sequences of the *CHS* genes by SWSSPROT (https://swissmodel.expasy.org/) and KOG (https://genome.jgi.doe.gov/Tutorial/tutorial/kog.html). According to the transcript sequences, a pair of specific primers (Table S1) was synthesized and used to amplify the ORF and genomic sequences of the *CHS* genes from the cDNA and the genomic DNA samples, respectively, by using Prime STAR HS DNA Polymerase (TaKaRa, China) with proof reading activity. The amplified products were purified by using Universal DNA Purification Kit (Tiangen, China), added dATP at the 3′ ends by using *Taq*^TM^ (TaKaRa, China), cloned into pMD19-T vector (TaKaRa, Japan), and sequenced at Sangon Biotech Co., Ltd (Shanghai, China). The sequencing results were aligned for gene structure on NCBI website (http://www.ncbi.nl-m.nih.gov), and predicted for putative proteins by using online tool SWISS-MODEL (https://swissmodel.expasy.org/). Phylogenetic analysis was conducted among the putative proteins by using MEGA7.0 software (https://www.megasoftware.net/).

### Function evaluation of the CHS genes

Three pairs of specific primers with appropriate recognition sites (Table S1) were used to amplify the ORF sequences without or with the termination codons from the harbored pMD19-T vectors by using Prime STAR HS DNA Polymerase (TaKaRa, Japan). The amplified products were purified as above, and inserted into transient expression vector pCAMBIA2300, prokaryotic expression vector pET-28a (+) and moncotyledonous expression vector pZZ00026, respectively, by using CloneExpress One Step Cloning Kit (Vazyme, China, Fig. S13).

The transient expression vectors pCAMBIA2300-*35S*-*CHS*-*eGFP* harboring the *CHS* genes of the two species, as well as the empty plasmid pCAMBIA2300-*35S*-*eGFP* (negative control), was infiltrated into the inner epidermis of onion. After incubation at 28 °C under dark for 24 h, the green fluorescence signal for subcellular localization was observed and photographed under fluorescence microscope (Olympus BX63, Japan)^45^.

The prokaryotic expression vectors pET-28a (+)−*CHS* were transformed into *E*. *coli*s train BL21 with freeze-thaw method. Until OD_600_ ≈ 0.6, the transformant cultures were added with IPTG to a final concentration of 0.5 mmol L^−1^, and incubated at 37 °C for 2 h. The heterologous expression of the *CHS* genes was detected by SDS-PAGE. The proteins were purified by using Ni-NTA Sefinose^TM^ Resin Kit (Sangon China), and determined for concentration by using NanoDrop™ One/OneC B-50Q (Thermo, USA). According to the manual (Table S2) of CHS enzyme activity kit (Genmed Scientifics Inc. USA), the purified protein and the IPTG-induced *E. coli* cells were reacted with 4-coumaric-CoA and 3 untis malonyl-CoA in the presence or absence of luteolin (a sensitive inhibitor of CHS), respectively. Along with the synthesis of chalcone, the released 4 units of CoA-SH with Ellman reagent 5,5-dithiobis (2-nitrobenzoic acid, DTNB) to produced yellow 5-thio-2-nitrobenzoic acid (TNB). The activity of chalcone synthase was quantitatively analyzed by the change of its absorption peak (412 nm wavelength). The *in vitro* and *in vivo* CHS enzyme activities were analyzed by the change of the absorption peak at 412 nm.

The moncotyledonous expression vector pZZ00026-*Ubi-CHS-T-nos* was mobilized into *Agrobacterium tumefaciens* strain EHA105, and used to transform rice calli of variety ZH11^46^. The transformed lines were identified by PCR amplification of a 1205 bp fragment of the *CHS* genes, and detected for content of total flavonoids by spectrophotography (described later).

### Expression analysis under inducing conditions

Two pairs of RT-qPCR primers (Table S1) was synthesized to amplify a 136 bp and a 221 bp fragment of the *CHS* ORFand the *Actin2* gene (used as internal reference), respectively, from the cDNA samples of the three replicates of each time point of the inducing treatments^47^. The amplification reaction was conducted by using SsoFastEvaGreenSupermix (Bio-Rad, USA) in Bio-Rad iCycler iQ5 RT-qPCR System. The 2^−ΔΔCT^ method of the CFX Manger™ software version 2.0 (Bio-Rad, USA) was used to normalize the expression differentiation between the internal reference and the *CHS* gene^48^. The relation expression levels under the inducing conditions were calculated by their expression level to those under the non-inducing control.

As described by Liu *et al*.^49^, six arbitrary degenerate primers (AD) and three nested primers (Table S1) complementary to the coding sequences of the *CHS* genes were synthesized, and used to amplify the promoter sequences of the *CHS* genes from the genomic DNA samples by TAIL-PCR. The products of the second and third rounds of amplification were separated by 1.2% argarose gel electrophoresis, purified and sequenced as above. The sequenced results were used for prediction of *cis*-affecting elements by Plant CARE software (http://bioinformatics.psb.ugent.be/webtools/plantcare/html/).

### Quantification of total flavonoids and free radical scavenging activity

The other leaf of each sample of 0 to 6 d of the induction was dried at 55 °C, ground to fine powder (extration quality), extracted in 95% alcohol in an ultrasonic instrument at 25 °C for 30 min. The extracts were filtered through Whatman No. 1 paper filter under reduced pressure (extraction volume). Referring to China national standardization for determination of total flavonoids in propolis^50^, 1 mL of each of the filtrates was added with 0.4 ml of 5% NaNO_2_, kept for 5 min, added with 0.4 ml of 10% Al(NO_3_)_3_, kept for 5 min, added with 4 ml of 4% NaOH for coloration, incubated at room temperature for 20 min, and determined for absorbency at 420 nm in a UV-1800 spectrophotometer (Shimadzu, Japan). The content of total flavonoids was calculated as:

$${\rm{Content}}\,{\rm{of}}\,{\rm{flavonoids}}=\frac{{{\rm{A}}}_{420}\times {\rm{V}}}{{\rm{m}}\times {\rm{d}}}$$

where, A_420_ were the absorbance at 420 nm, V were total volume of the extract, m were the extration quality from the leaf of each sample (1 g), d were the dilution multiple.

As described by Sharma and Bhat^51^, 2 mL of each of the filtrates, as well as two milliliters of ethanol (blank control), were added with 2 ml of 0.04 mmol/L ethanol solution of 1-diphenyl-2-picrylhydrazyl (DPPH), incubated in dark for 20 min, and detected the absorbance at 517 nm in an UV-1800 spectrophotometer. The free radical scavenging activities were calculated as:

$${\rm{Free}}\,{\rm{radical}}\,{\rm{scavenging}}\,{\rm{activity}}=\frac{1-({{\rm{A}}}_{{\rm{i}}}-{{\rm{A}}}_{{\rm{b}}})}{{{\rm{A}}}_{{\rm{n}}}}\times 100$$

where, A_i_, A_b_ and A_n_ were the absorbance of the induced samples, the non-inducing samples and the blank control.

### Quantification of flavonalconstituents and anthocyanidin

Ten microliters of each of the filtrates were loaded into HPLC (Agilent 1260) equipped with Brownlee SPP C18 column (E-Merck, 2.7 μm, 4.6 × 150 nm). Gradient elution was conducted with 80% mobile phase A (98% H_2_O and 2% H_3_PO_4_) and 20% mobile phase B (CH_3_Cl) for 5 min, 55% mobile phase A and 45% mobile phase B for 43 min, and 80% mobile phase A and 20% mobile phase B for 2 min at a flow rate of 1 ml/min and column temperature of 30 °C. The flavonal constituents (rutin, quercetin and kaempferol) were monitored at 360 nm and identified by comparison of retention time with their authentic samples. Their relative contents were quantified and expressed as percentage peak area.

As described by Tanaka *et al*.^52^, one leaf of each sample of 0 (control), 1, 2, 3, 4, 5 and 6 d of the induction, was pulverized to fine powder in liquid nitrogen, extracted with acidified (1% HCl) methanol in dark with shaking for 48 h, and centrifuged at 4000 g for 10 min. The supernatant was used to determine for absorbance at 535 nm in an UV-1800 spectrophotometer. The anthocyanidin content was indicated by absorption value.

### Determination of MDA content

As described by Yu *et al*.^53^, the other leaf of the each sample of 0 (control), 1, 2, 3, 4, 5 and 6 d of the induction was pulverized with 10% trichloroacetic acid (TCA) and centrifuged at 4000 g for 10 min. Two milliliters of the supernatant was added with 2 mL of 0.06% thiobarbiuricacid (TBA), and bathed in boiling waterfor 10 min. After cooled, the samples were centrifuged at 7000 g for 5 min. The supernatant was determined for absorbance at 450, 532, and 600 nm, respectively, in an UV-1800 spectrophotometer. The MDA content was calculated as:

$${\rm{MDA}}\,{\rm{content}}=[6.452\times ({{\rm{A}}}_{532}\mbox{--}{{\rm{A}}}_{600})-0.559\times {{\rm{A}}}_{450}]\times [{\rm{V}}\div({\rm{V}}\mbox{'}\times {\rm{W}})]$$

where, A_450_, A_532_, and A_600_ were absorbance at 450, 532, and 600 nm, respectively. V,V’, and W were total volume of the extract, the determined volume (2 mL), and the fresh weight of the sample.

## Supplementary information

Supplementary Information
