# Supplementary material for: Expression response of chalcone synthase gene to inducing conditions and its effect on flavonoids accumulation in two medicinal species of Anoectochilus
Source: Sci Rep. 2019 Dec 27;9:20171. doi: 10.1038/s41598-019-56821-0 (PMC6934732; doi:10.1038/s41598-019-56821-0)
Supplement: Supplementary file 1 — Supplementary Information [file 41598_2019_56821_MOESM1_ESM.docx]

Expression response of chalcone synthase gene to inducing conditions and its effect on flavonoids accumulation in two medicinal species of *Anoectochilus*

Lin Yang^a, b^, Jun Cheng Zhang^b^,Jing Tao Qu^a^, Gang He^c^,Hao Qiang Yu^a^, Wan Chen Li^a, *^, Feng Ling Fu^a, *^

a Maize Research Institute, Sichuan Agricultural University, Chengdu, Sichuan 611130, PR China

b Medical Plant Exploitation and Utilization Engineering Research Center, Fujian Province University, Sanming University, Sanming 365004, People’s Republic of China

c Key Laboratory of Medicinal and Edible Plants Resources Development of Sichuan Education Department，Sichuan Industrial Institute of Antibiotics, Chengdu University, Chengdu 610052, PR China

*Corresponding authors:

Maize Research Institute, Sichuan Agricultural University, Chengdu, Sichuan 611130, PR China

WanChen Li, E-mail address: aumdyms@sicau.edu.cn

FengLing Fu, E-mail address: ffl@sicau.edu.cn


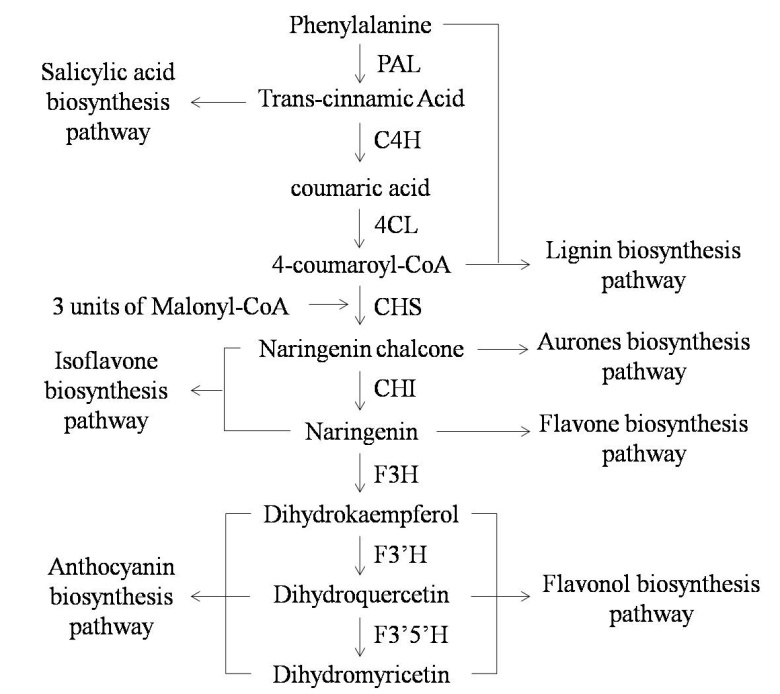


**Supplemental figure 1. Flavonoids biosynthesis of phenylpropanoid pathway in plant.**


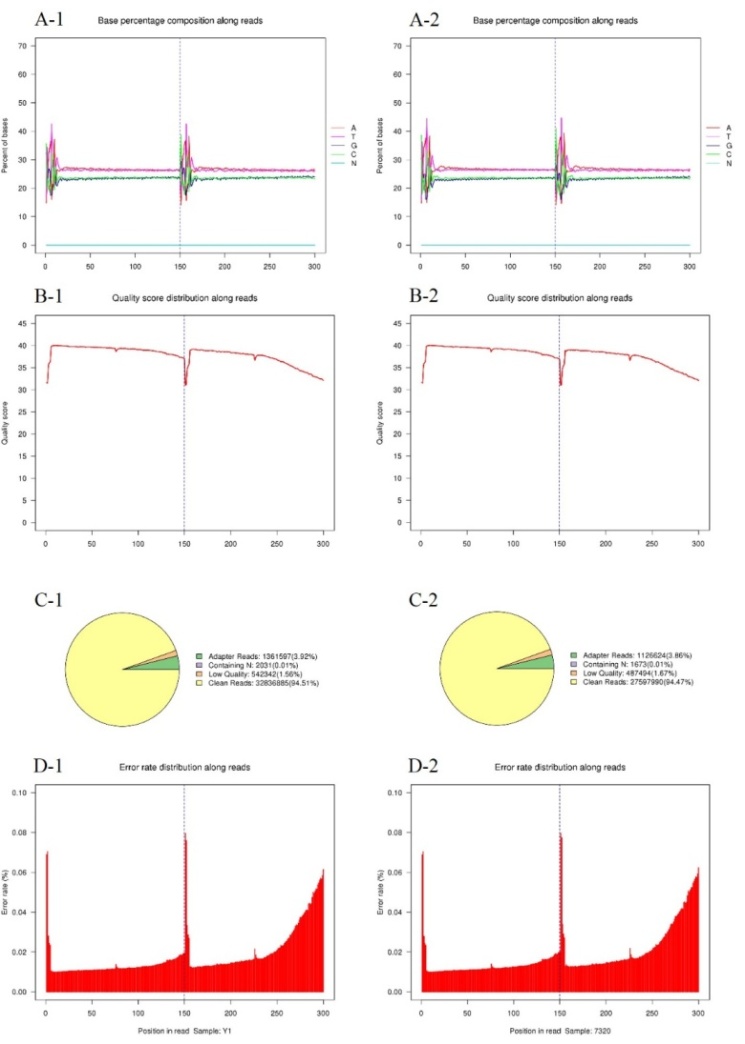


**Supplemental figure 2. RNA-seq throughput and quality of *A. roxburghi* and *A. formosanus*.** 1: *A. formosanus*, 2: *A. roxburghii*. (A) base percentage composition along read. (B) quality score distribution along reads. (C) rate of clean reads. (D) error rata distribution along reads.


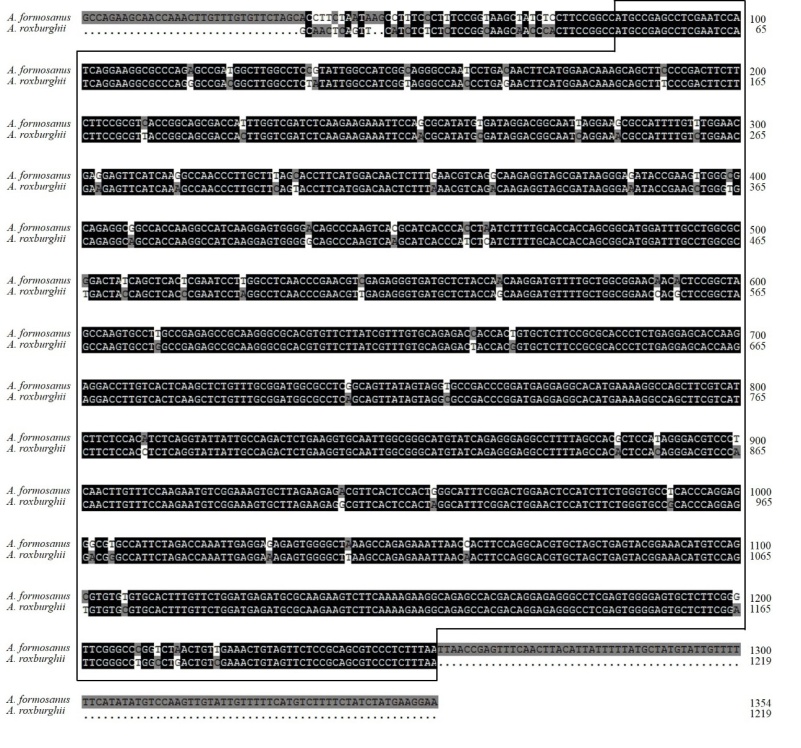


**Supplemental figure 3. Alignment between transcript sequences of *CHS* gene of *A. formosanus* and *A. roxburghii*.** The ORF sequences were framed.


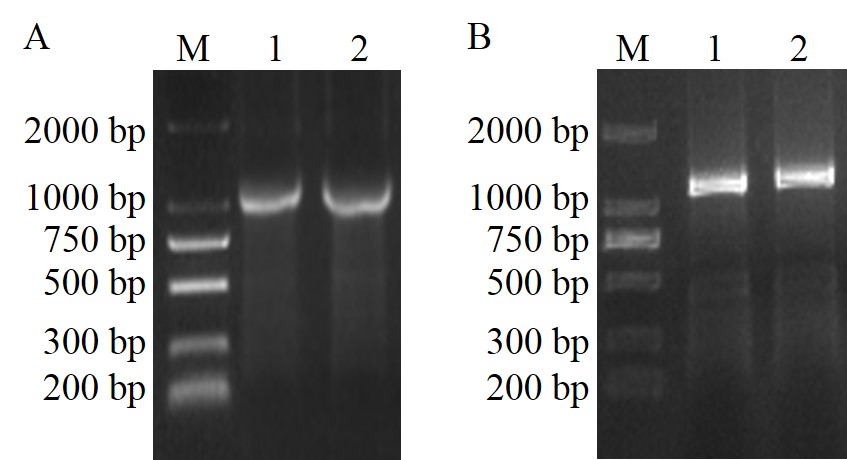


**Supplemental figure 4. The fragments of the *CHS* gene from cDNA and DNA in *A. formosanus* and *A. roxburghii.*** M: DNA molecular maker DL2000; 1: The fragments of the *PAL* gene from *A. formosanus*; 2: The fragments of the *PAL* gene from *A. roxburghii*. (A) The fragments of the *PAL* gene from cDNA. (B) The fragments of the *PAL* gene from DNA.


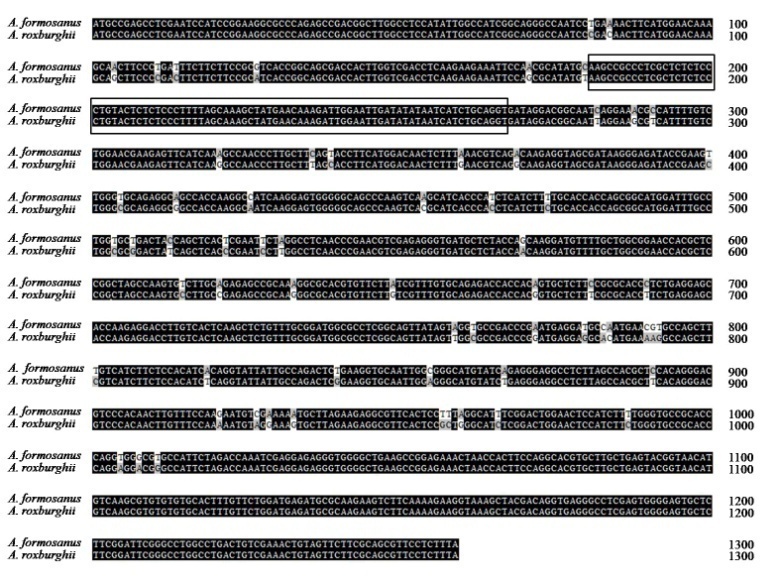


**Supplemental figure 5. Genomic sequences of the *CHS* genes between *A. formosanus* and *A. roxburghii*.** The black, gray and white backgrounds denote similarity of 100%, 66.6% and 0%, respectively, and the frame represents the introns.


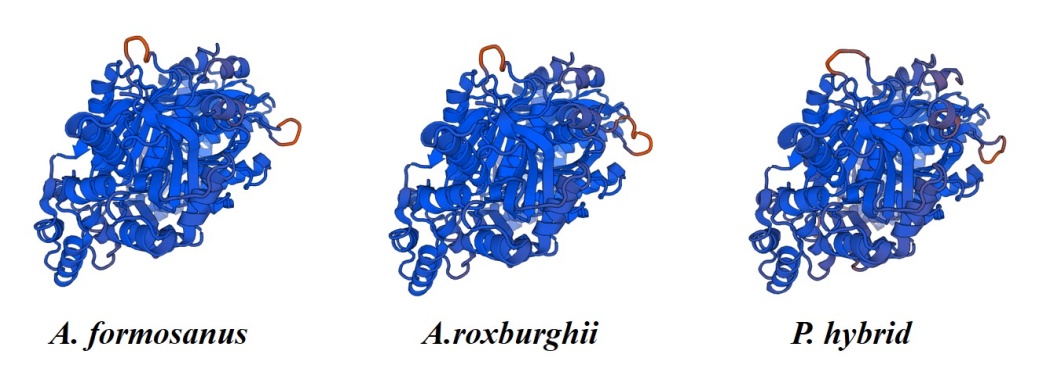


**Supplemental figure 6. Predicted three-dimensional models of the putative proteins of the *CHS* genes between *A. formosanus*, *A. roxburghii* and *P. hybrida*.**


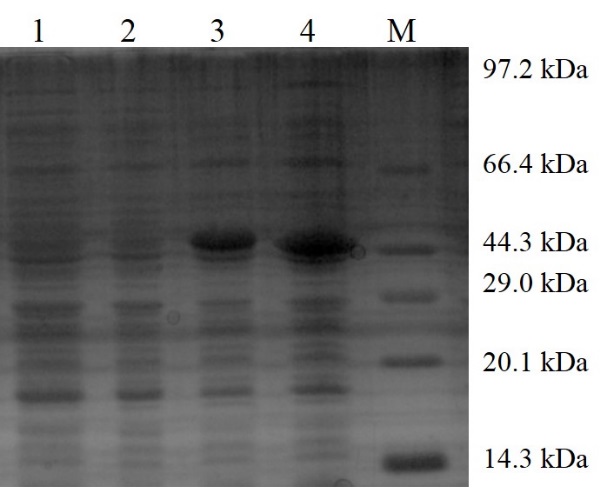


**Supplemental figure 7. Heterologous expression of *CHS* gene separated by SDS-PAGE.** M: Protein molecular weight marker; 1: uninduced transformant of the *CHS* gene from *A. formosanus*; 2: uninduced transformant of the *CHS* gene from *A. roxburghii*; 3: IPTG-induced transformant of the *CHS* gene from *A. formosanus*; 4: IPTG-induced transformant of the *CHS* gene from *A. roxburghii*.


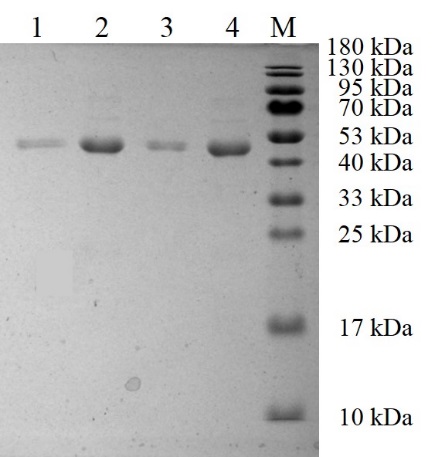


**Supplemental figure 8.** **Purified CHS protein separated by SDS-PAGE.** M: Protein molecular weight marker; 1: uninduced transformant of the *CHS* gene from *A. formosanus*; 2: uninduced transformant of the *CHS* gene from *A. roxburghii*; 3: IPTG-induced transformant of the *CHS* gene from *A. formosanus*; 4: IPTG-induced transformant of the *CHS* gene from *A. roxburghii*.


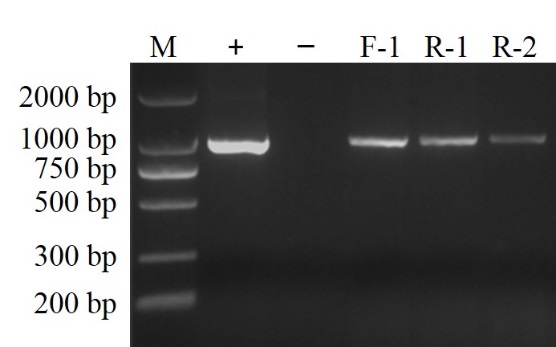


**Supplemental figure 9.** **PCR detection of transgenic lines.** M: DNA molecular maker DL2000; +: positive control (expression vector pZZ00026-*Ubi-CHS-T-nos*); -: negative control (untransformed acceptor line); F-1: T_1_ line transformed by the *CHS* gene from *A. formosanus*; R-1 and R-2: T_1_ lines transformed by the *CHS* gene from *A. roxburghii*.

**
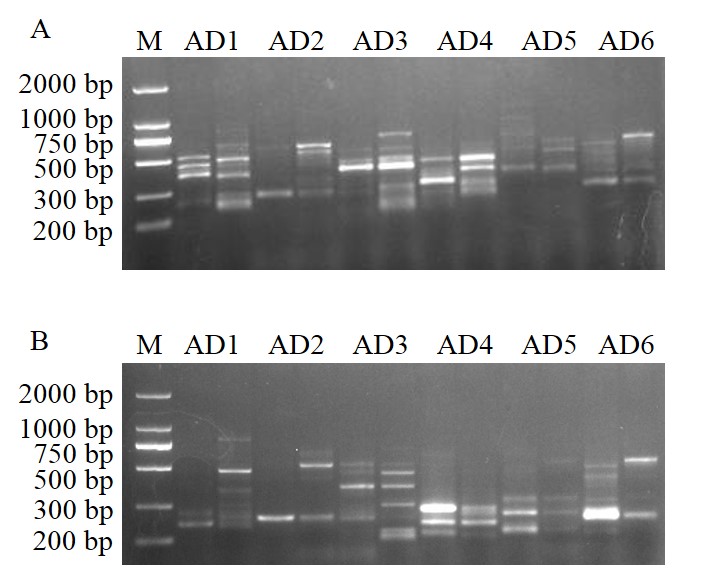
**

**Supplemental figure 10. Specific fragments amplified from the genomic DNA samples of *A. formosanus* (A) and *A. roxburghii* (B) in the second and third rounds of TAIL-PCR amplification.** M: DNA molecular marker DL200; AD1-AD6: arbitrary degenerate primers.

**
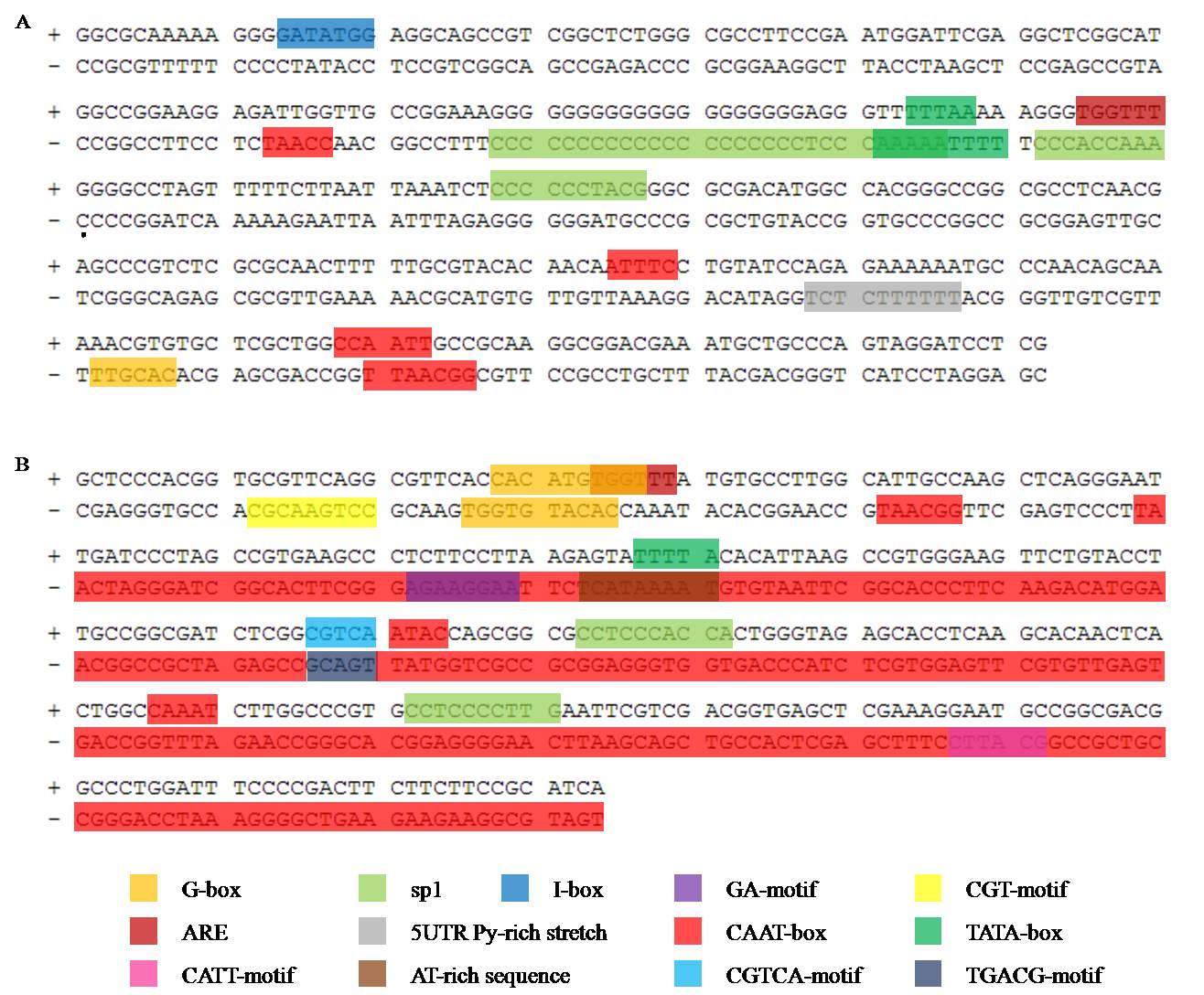
**

**Supplemental Figure S11. The *cis*-acting elements during promoter sequence of gene *CHS* of *A. formosanus* and *A. roxburghii*.** (A) cis-acting elements during promoter sequence of gene CHS of *A. formosanus*. (B) cis-acting elements during promoter sequence of gene *CHS* of *A. roxburghii*.

**
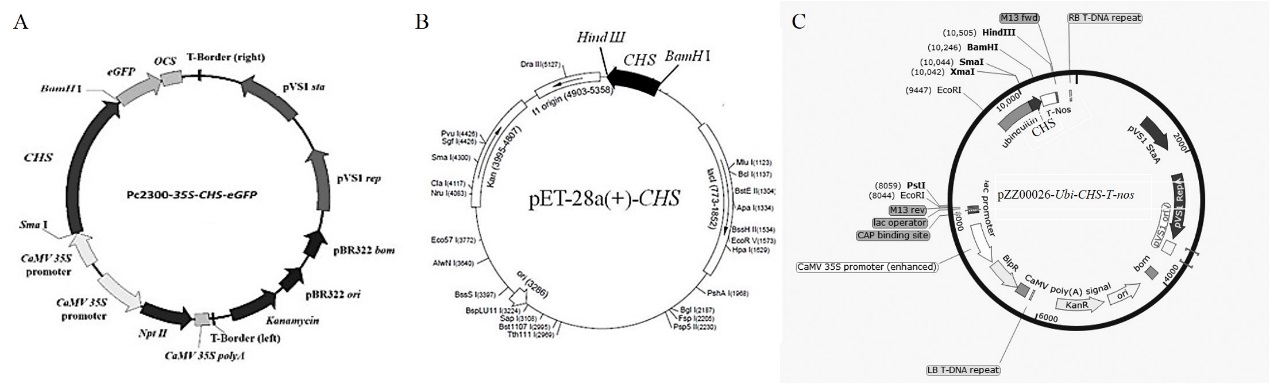
**

**Supplemental figure 12. The expression vector of the *CHS* gene of *A. formosanus* and *A. roxburghii*.** (A) The transient expression vector pCAMBIA2300-*CHS*-*eGFP*. (B) The protokaryotic expression vector pET-28a(+)-*CHS*. (C) The moncotyledonous over expression vector pZZ00026-*Ubi*-*CHS*-*T-nos*.

**Supplemental table 1. PCR primers**

| Use | | Sequence (5´-3´) | | Restriction site | |
| --- | --- | --- | --- | --- | --- |
| Cloning of *CHS* gene | | ATGCCGAGCCTCGAATCCA | |  | |
|  |  | TTAAAGAGGAACGCTGCGAA | |  | |
| Construction of transient vector pCAMBIA2300-*35S*-*CHS*-*eGFP* | | catttggagaggacagggtacccgggATGCCGAGCCTCGAATCCA | | *Sma* I (underlined) | |
|  |  | tcgcccttgctcaccatggtactagtAAGAGGAACGCTGCGAA | | *Spe* I (underlined) | |
| Construction of protokaryotic vector pET-28a(+)-*CHS* | | tggacagcaaatgggtcgcggatccATGCCGAGCCTCGAATCCA | | *Bam*H I (underlined) | |
|  |  | ggtgctcgagtgcggccgcaagcttTTAAAGAGGAACGCTGCGAA | | *Hind* I (underlined) | |
| Construction of moncotyledonous vector pZZ00026-*Ubi-CHS-T-nos* | | gtttggtgttacttcccgggATGCCGAGCCTCGAATCCA | | *Sma* I (underlined) | |
|  |  | gccaaatgtttgaacgatcggatccTTAAAGAGGAACGCTGCGAA | | *Bam*H I (underlined) | |
| Amplification detection of transgenic lines | | GCTCACCCTGTTGTTTGGTG | |  | |
|  |  | AAGAGGAACGCTGCGAAGAA | |  | |
| RT-qPCR | *CHS* gene | AGGCGTTCACTCCTTTAGGC | |  | |
|  |  | ACGTGCCTGGAAGTGGTTAG | |  | |
|  | *Actin2* gene | CGGGCATTCACGAGACCAC | |  | |
|  |  | AATAGACCCTCCAATCCAGACACT | |  | |
| TAIL-PCR | Nested primer | ACTCTTCGTTCCAGACAAAATGAC | |  | |
|  |  | TGATGCGGAAGAAGAAGTCGGGGAA | |  | |
|  |  | CATGAAGTTGTCGGGATTGGCCCTG | |  | |
|  | Arbitrary degenerate primer | NTCGASTWTSGWGTT | Where, S = G/C, W = A/T, N = A/T/C/G. | | |
|  |  | NGTCGASWGANAWGAA |  |  |  |
|  |  | NGTASASWGTNAWCAA |  |  |  |
|  |  | STTGNTASTNCTNTGC |  |  |  |
|  |  | AGWGNAGWANCAWAGG |  |  |  |
|  |  | TGWGNAGWANCASAGA | | |  |

Supplemental Table 2 The manual of CHS enzyme activity kit

| step | content |
| --- | --- |
| 1 Assay preparation | 1. Turn on the spectrophotometer (UV752) with a wavelength of 412 nm at 30 ℃. 2. Read it every 5 minutes for 7 times (30 minutes) and then set it to zero. 3. Collect the bacteria by centrifugation at 8000 g for 10 minutes, and re-suspend the combined buffer [25 mmol/L Tris-HCl (pH 8.0), 0.5 mol/L NaCl, 5 mmol/L imidazole]. 4. Put into the pre-cooled homogenizer and homogenize the bacterial fluid in ice-water mixture for 40 minutes. |
| 2 Blank determination(Ablank) | 1. Mix 205 µL buffer solution (Reagent C) and 25 µL substrate solution (Reagent E) in a new cuvette at 30 ℃ for 3 minutes. 2. Add 20 µL extracting solution or 200 µg protein, 3. Determine for absorbency immediately at 412 nm in a UV752 spectrophotometer for 7 times (30 minutes), as a control. |
| 3 Determination of total enzyme activity(A total) | 1. Mix 185 µL buffer solution (Reagent C), 25 µL reaction solution (Reagent D) and 25 µL substrate solution (Reagent E) at 30 ℃ for 3 minutes. 2. Add 200 µg protein or 20 µL homogenized bacterial fluid, and mix them in 3 seconds. 3. Determine for absorbency immediately at 412 nm in a UV752 spectrophotometer for 7 times (30 minutes). |
| 4 Determination of non-specific enzyme activity(A non-specific) | 1. Mix 20 µL specific solution (Reagent F) and 20 µL extracting solution or 200 µg protein at 30 ℃ for 30 minutes. 2. Mix 160 µL buffer solution (Reagent C), 25 µL reaction solution (Reagent D) and 25µL substrate solution (Reagent E) at 30 ℃ for 3 minutes. 3. Add 40 µL liquid in step a, and mix them in 3 sec. 4. Determine for absorbency immediately at 412 nm in a UV752 spectrophotometer for 7 times (30 minutes). |
| 5 Calculation of enzyme activity | 1. The total CHS enzyme activity =[(A total-Ablank) × V × d] ÷[m×13.6×t×4] 2. The non-specific CHS enzyme activity =[(A non-specific -Ablank) × V × d] ÷[m×13.6×t×4] 3. The specific CHS enzyme activity = The total CHS enzyme activity - The non-specific CHS enzyme activity |

Where, V was total volume (the *in vitro* CHS enzyme activity using 250 µL, and the *in vivo* CHS enzyme activity useing 270 µL), d was the dilution multiple, m was the protein quality (the *in vitro* CHS enzyme activity using 200 µg, and the *in vivo* CHS enzyme activity useing a fixed value), 13.6 was millimolar absorption coefficient, t was the time (30 min), 4 was 4 units of CoA-SH of in a reaction.
